# Cross-attention multi-branch CNN using DCE-MRI to classify breast cancer molecular subtypes

**DOI:** 10.3389/fonc.2023.1107850

**Published:** 2023-03-07

**Authors:** Liang Sun, Haowen Tian, Hongwei Ge, Juan Tian, Yuxin Lin, Chang Liang, Tang Liu, Yiping Zhao

**Affiliations:** ^1^ The College of Computer Science and Technology, Dalian University of Technology, Dalian, Liaoning, China; ^2^ Department of Radiology, The Second Affiliated Hospital of Dalian Medical University, Dalian, Liaoning, China

**Keywords:** breast cancer, molecular subtypes, deep learning, attention mechanism, MRI

## Abstract

**Purpose:**

The aim of this study is to improve the accuracy of classifying luminal or non-luminal subtypes of breast cancer by using computer algorithms based on DCE-MRI, and to validate the diagnostic efficacy of the model by considering the patient’s age of menarche and nodule size.

**Methods:**

DCE-MRI images of patients with non-specific invasive breast cancer admitted to the Second Affiliated Hospital of Dalian Medical University were collected. There were 160 cases in total, with 84 cases of luminal type (luminal A and luminal B and 76 cases of non-luminal type (HER 2 overexpressing and triple negative). Patients were grouped according to thresholds of nodule sizes of 20 mm and age at menarche of 14 years. A cross-attention multi-branch net CAMBNET) was proposed based on the dataset to predict the molecular subtypes of breast cancer. Diagnostic performance was assessed by accuracy, sensitivity, specificity, F1 and area under the ROC curve (AUC). And the model is visualized with Grad-CAM.

**Results:**

Several classical deep learning models were included for diagnostic performance comparison. Using 5-fold cross-validation on the test dataset, all the results of CAMBNET are significantly higher than the compared deep learning models. The average prediction recall, accuracy, precision, and AUC for luminal and non-luminal types of the dataset were 89.11%, 88.44%, 88.52%, and 96.10%, respectively. For patients with tumor size <20 mm, the CAMBNET had AUC of 83.45% and ACC of 90.29% for detecting triple-negative breast cancer. When classifying luminal from non-luminal subtypes for patients with age at menarche years, our CAMBNET model achieved an ACC of 92.37%, precision of 92.42%, recall of 93.33%, F1of 92.33%, and AUC of 99.95%.

**Conclusions:**

The CAMBNET can be applied in molecular subtype classification of breasts. For patients with menarche at 14 years old, our model can yield more accurate results when classifying luminal and non-luminal subtypes. For patients with tumor sizes ≤20 mm, our model can yield more accurate result in detecting triple-negative breast cancer to improve patient prognosis and survival.

## Introduction

Breast cancer is one of the most prevalent cancers in women and one of the main causes of cancer-related death in women under the age of 45. There are nearly 410000 patients who die of breast cancer annually all over the world (1, 2). Breast cancer is highly heterogeneous. The different molecular subtypes of breast cancer are significantly different in treatment, radiochemotherapy sensitivity, and prognosis (3, 4). Luminal type A breast cancer subtype well responds to endocrine therapy, has a low risk of recurrence and metastasis, and has a good prognosis. Luminal type B well responds to endocrine therapy, but is more proliferative than luminal type A and may easily recur in the early stages (5). HER2-positive and triple-negative subtypes have a high malignancy grade and poor prognosis (6). Meanwhile, HER2-positive well responds to targeted molecular therapy. Therefore, it is important to distinguish between luminal and non-luminal breast cancer for accurate treatment.

Molecular typing of breast cancer mainly depends on immunohistochemical examination of biopsy specimens. Histopathological examination is not only invasive, time-consuming, and expensive, but also easily leads to infection, hematoma, and other complications. Because of the great heterogeneity of the tumor, the biopsy tissue cannot fully represent the biological behavior of the tumor. MRI has the advantages of being non-invasive, resolving soft tissue well, and non-radiation, and it has unique advantages for breast examination. The studies show that MRI imaging features are helpful in identifying molecular subtypes of breast cancer. luminal A and luminal B masses are irregularly shaped and have burr-like edges (7). Triple-negative breast cancer usually shows a well-defined round mass with annular enhancement (8). Therefore, the prediction of molecular subtypes of breast cancer based on MRI features can effectively reduce the number of biopsies, alleviate the pain of patients, reduce the burden on patients, and provide a reference for individualized treatment.

However, predicting the molecular subtype of a tumor based on the MRI features of breast cancer is difficult because of two issues: (1) low contrast between the lesion area and normal tissue; (2) In clinical practice, the shapes of different subtypes of tumors are very similar, and the interpretation results of professional physicians are greatly influenced by the subjective factors of the physicians. So it is difficult to distinguish the molecular subtypes of breast cancer from the naked eye.

Many traditional machine learning algorithms have been applied to relevant breast cancer analysis tasks (9–13). However, these traditional machine learning methods rely on manual feature extraction with strong a priori, poor model generalization, and low robustness, which are difficult to find discriminating features manually and solve the classification of breast cancer subtypes. In recent years, with the development of deep learning technology, deep learning algorithms have been widely used in medical image processing, such as tumor detection (14–16) and segmentation (17–19), benign and malignant differentiation (20–23), etc. A lot of work (24–27) has been devoted to the problems related to breast cancer subtype classification, They try to extract discriminative features of breast cancer MRI by using deep learning models. Their experimental results illustrate the feasibility of predicting the molecular subtypes of tumors based on the MRI features of breast cancer.

At the same time, because these models are only direct applications or simple modifications of existing models, they do not make targeted measures to address the above-mentioned problems in breast cancer subtype classification. Therefore, these models cannot distinguish well between the different molecular subtypes of breast cancer. So to solve the above issues and improve the performance of breast cancer subtype classification based on breast MRI images, we propose a new deep network model CAMBNET to extract high-level feature information and focus on lesion objects. The model includes a multi-branch module, a cross-attention mechanism, and a deep feature extraction module. Specifically, using only a single branch for feature extraction may not be effective and the multi-branch module is used to extract richer features, In response to the problem of low contrast between lesions and normal areas in the data set and the very similar shape of lesions in different diseases, the attention mechanism has been widely used in similar problems. Therefore, we designed the cross-attention module to help the network pay attention to salient objects, and as the depth of the model increases, the model can extract deeper features and further improve the feature extraction capability of the model. So the deep feature extraction module is used to further extract the deep features. Due to many limitations such as the rarity of the disease and the lack of appropriately labeled medical expertise, resulting in a relatively small dataset for breast cancer subtype classification, we chose a smaller depth of model layers and constructed our deep learning model.

Studies have shown that early menarche age increases the risk of luminal-type breast cancer, which may be related to endogenous estrogen exposure (28). Tumor size is one of the indices to evaluate the staging of breast cancer and has an important significance for the selection of surgical methods. And small tumors offer limited imaging options, which can easily lead to misdiagnosis. The earlier the age of menarche, the higher the rate of axillary lymph node metastasis and the worse the prognosis of breast cancer patients (29). Therefore, the initial aim of this study was to develop a new deep network model for predicting luminal and non-luminal subtypes of breast cancer using DCE-MRI images. We also investigated the diagnostic efficacy of different age groups for menarche (≤ 14 years and >14 years) and tumor size groups (≤ 20 mm and >20 mm).

## Materials and methods

### Data collection

This is a retrospective study and is approved by the Second Affiliated Hospital of Dalian Medical University Ethics Committee. Non-specific invasive breast cancer patients admitted to our hospital from May 2017 to December 2019 were selected. The inclusion criteria consisted of: (1) patients with non-specific invasive breast cancer confirmed by biopsy or surgical pathology had complete immunohistochemical results and molecular subtypes were identified. (2) DCE-MRI was performed within a week before the operation. (3) complete clinical data, including age and menstrual status. The exclusion criteria consisted of (1) percutaneous biopsy or neoadjuvant chemotherapy or radiotherapy before MRI examination or (2) tumor was inconclusive because of artifacts or no visible region of interest (ROI) or (3) image quality was poor or (4) molecular typing of immunohistochemical data of pathologic diagnosis was incomplete. Ultimately, 160 patients with breast cancer were enrolled in the study, including 84 with luminal subtypes (luminal A and luminal B) versus 76 with non-luminal subtypes (HER2-positive and triple-negative).

### MRI technique

Images were obtained with a 3.0-T magnetic resonance imaging scanner (Discovery 750W, GE). A special coil was used to scan the breast. Patients were in the prone position with the head tilted forward and the double breasts naturally suspended in the coil. T1WI, T2WI, DWI and 3D volume images of the breast (3D VIBRANT) were performed. The 3D VIBRANT scan parameters are as follows: TR 7.6 ms, TE 3.8 ms, layer thickness 1.2 mm, FOV 320 mm × 320 mm, flip angle 15°, matrix 288 × 288. The contrast agent was injected into the antecubital vein through a high-pressure injector (GE Company, USA). The flow rate of the contrast agent was 2 mL/s and the dose was 0.2 mmol/kg. After injection, 7 consecutive non interval scans were performed, each scan lasting 1 minute and 7 seconds.

### Immunohistochemical examination

The receptors ER, PR, HER2, and Ki-67 were detected by immunohistochemistry. (1) ER/PR positivity was defined as ≥1% positive staining of tumor nuclei (2) HER-2 positivity was defined as Her-2 (3+), (-, and (1+) were defined as HER -2 negativity. Fluorescence *in situ* hybridization (FISH) was used to detect HER -2 fluorescence *in situ* hybridization (3) Ki-67 showed high expression (≥14%) and low expression (<14%). Breast cancer is classified into four subtypes according to receptor status, defined as follows: (1) Luminal A: ER and/or PR positive, HER -2 negative and KI-67 low expression (2) Luminal B: ER and/or PR positive, Ki-67-high expression, Her-2(positive or negative) (3) Her-2 positive: ER, PR negative, HER -2 positive (4) triple-negative: ER, PR, Her-2 negative.

### Image processing

Region of interest (ROI) outlining the tumor region in MRI T1WI, T2W1, and DCE (selected third-stage enhanced images after contrast injection) by 2 senior diagnostic breast MRI physicians should include all tumor regions, including cystic and necrotic regions.

**Figure 1:**
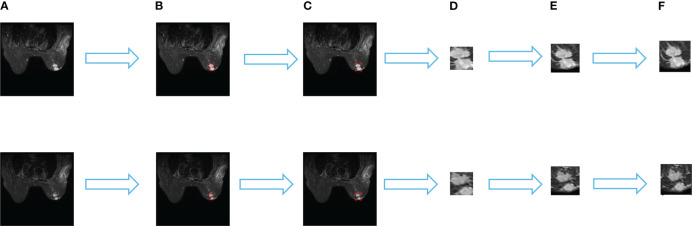
Data set processing process. **(A)** is the original image, **(B)** is the specific outline of the tumor sketched by the physician, **(C)** is the four edge points in the specific outline, **(D)** is the minimum matrix covering the tumor, **(E)** shows that 10 pixels are added to each direction of the four points to crop the outline area. and **(F)** is the cropped image uniformly resized to 64×64 pixels by a bilinear interpolation method.

As the physician outlines the specific contour of the tumor, we derive the minimum matrix covering the tumor by extracting the most marginal points in the four directions of the contour. These four points were added 10 pixels in their respective directions to crop their contour areas, and the cropped images were uniformly adjusted to 64 × 64 pixels by bilinear interpolation. And the image is normalized by transforms. Normalize. The specific process is shown in **Figure 1**. 

### Data set partitioning

Breast images from 20% of the cases in the dataset were used as the test dataset, and 80% were kept as the training dataset while ensuring that no patient images appeared in both the training and test sets. The number of each sub-dataset in the dataset is shown in **Table 1**.

**Table 1:** The number of each sub-dataset in the dataset.

Molecular subtypes	cases			
	Size		Age	
	≤ 20	>20	≤ 14	>14
Luminal A	20	18	22	16
Luminal B	27	20	30	17
HER2+	19	28	21	26
Triple-negative	7	21	10	18
Total	73	87	83	77

### Deep learning model

**Figure 2:**
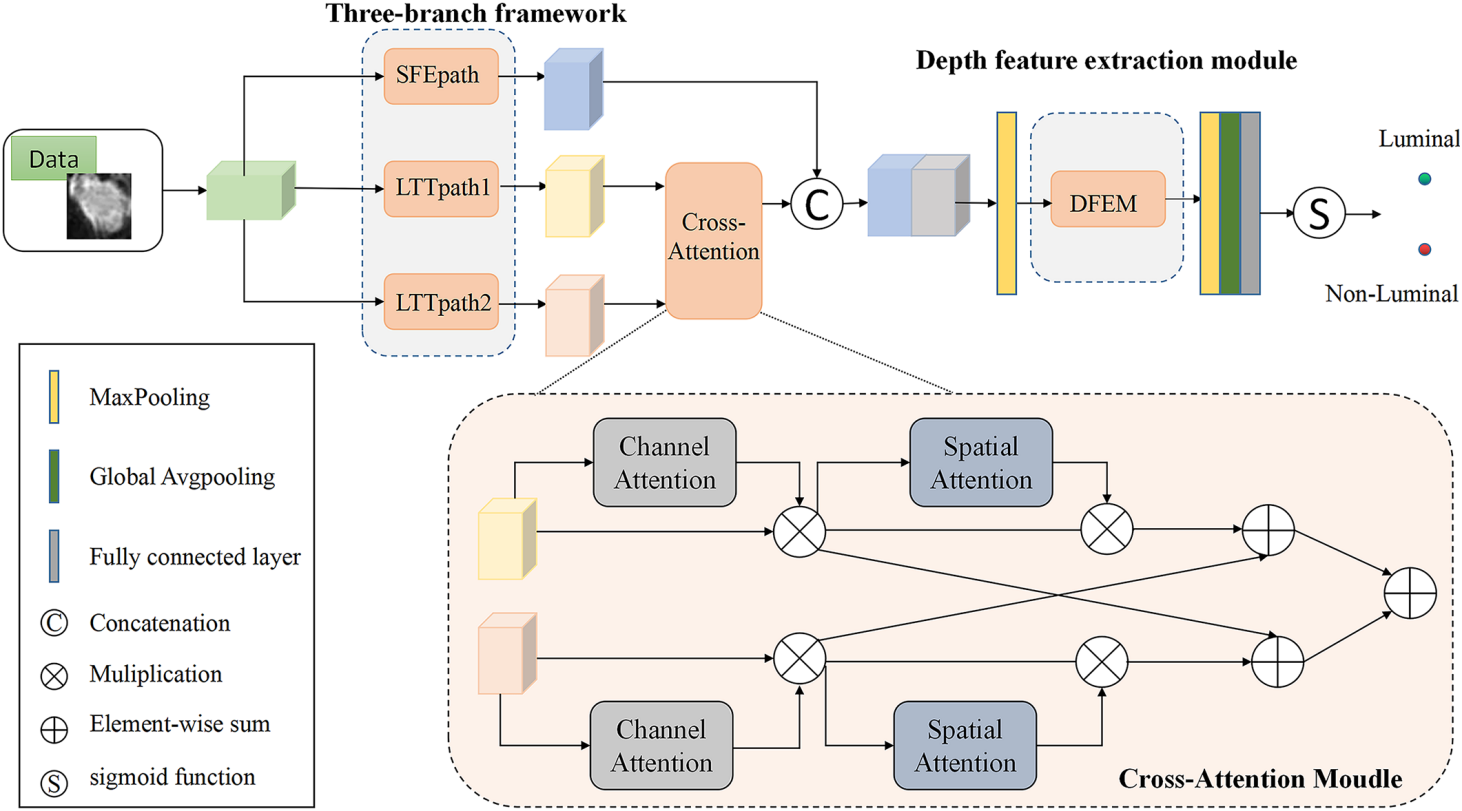
Structure of the model (SFEpath, Shallow feature extraction path; LTTpath, Locating the tumor path; DFEM, Depth Feature extraction module.).

In this paper, a multi-branch crossover network is proposed to extract high-level features. Two of the branches fuse the extracted features after passing through the cross-attention module, and then the fused features are fused with the shallow features extracted by SFEpath to improve the classification performance of MRI images for two different subtypes. The proposed framework is shown in **Figure 2**. From **Figure 2**, we can see that our proposed network architecture consists of three main parts: the three-branch framework, the cross-attention module, and the deep feature extraction module. The specific model parameters are shown in **Table 2**. We will explain these modules in detail in the following sections.

**Table 2:** Parameter configurations of different modules.

Input layer	Input layer dimensions	Filter size	Channels	Output layer
Pre_layer	64 × 64 × 3	3 × 3	16	Three-branch structure
SFEpath				
Conv1_1	64 ×64×16	1 × 1	128	Dwconv1_2
Dwconv1_2	64 ×64 ×128	3 × 3	128	SEB1_3
SEB1_3	64 ×64 ×128	——	——	Conv1_4
Conv1_4	64 ×64 ×128	1 × 1	16	DFEM
LTTpath1				
Conv2_1	64 ×64×16	1 × 1	8	Dwconv2_2
Dwconv2_2	64 ×64×8	3 ×3	8	Conv2_3
Conv2_3	64 ×64×8	1 × 1	16	Cross_Attention
Cross_Attention	64 ×64×16	——	——	DFEM
LTTpath2				
Conv3_1	64 ×64×16	1 × 1	4	Conv3_2
Conv3_2	64 ×64×4	1 × 3	8	Conv3_3
Conv3_3	64 ×64×8	3 × 1	8	Conv3_4
Conv3_4	64 ×64×8	1 × 3	8	Conv3_5
Conv3_5	64 ×64×8	3 × 1	16	Cross_Attention
Cross_Attention	64 ×64×16	——	——	DFEM
DFEM				
Maxpool4_1	64 ×64×32	——	——	Conv4_2
Conv4_2	32 ×32×32	1 × 1	128	Dwconv4_3
Dwconv4_3	32 ×32 ×128	3 × 3	128	SEB4_4
SEB4_4	16 ×16 ×128	——	——	Conv4_5
Conv4_5	16 ×16 ×128	1 × 1	64	Maxpool4_6
Maxpool4_6	16 ×16 ×64	——	——	Fc4_7
Fc4_7	8 ×8 ×64	64 × 2	——	output

### Three-branch structure

**Figure 3:**
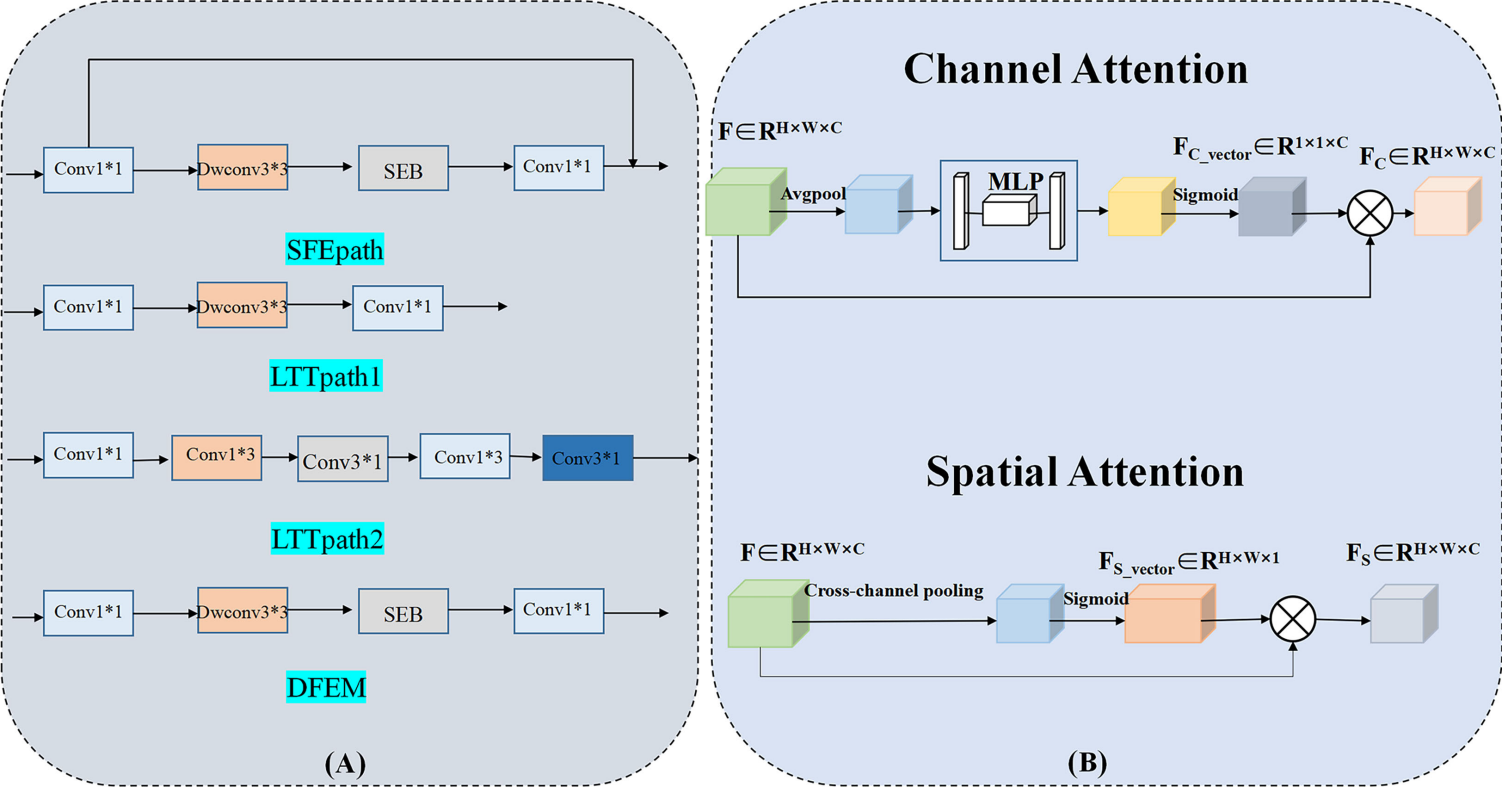
Three-branch structure and Cross-attention Module. (Conv, Convolutional layer; Dwconv, Depthwise convolutional layer; SEB, Squeeze-and-Excitation Block).

Due to the limited amount of data in the dataset, an overly complex model is too easy to over-fit. So three light branching paths are designed. From **Figure 3A**, SFB refers to the Squeeze-and-Excitation module (30) and SFEpath is added to the branching framework to extract depth features as the input of depth concern, and part of the original input feature information is directly transferred to the output features by using the residual connection. The residual connection can simplify the difficulty of feature learning, protect the integrity of feature information to a certain extent, and alleviate the problem of model degradation in deep networks. It enables the model to better learn the shallow information such as the texture and shape of the breast image and makes the features extracted by the model richer.

Since the contrast between tumor and background is low, a network capable of extracting multiple depth features from different branches is needed. To obtain more depth features, a multi-branch network was designed using its two branches (called LTTpath1 and LTTpath2), inspired by the Inception model. That is, the main structure of this network uses asymmetric rotating *c*_1_×*n* and *n*×*c* ^1^ filters to reduce the parameter size and computational cost, instead of the traditional *n*×*n*. The two main effects are (1) the downscaling of the data and the introduction of more nonlinearities and (2) the improvement of the generalization.

### Cross-attention Module

For the model to learn specific differences and relationships between different subtypes of breast cancer, the interference of irrelevant regions is suppressed. We propose a cross-attention mechanism that focuses on the salient features of each breast cancer subtype. Our proposed cross-attention module consists of a spatial attention module and a channel attention module, as shown in **Figure 3B**, where the spatial attention module and the channel attention module are single-path modules instead of the dual-path module of CBAM because in breast cancer subtype classification experiments we found that the combination of single-path with cross-path patterns is better than the combination of dual-path patterns or dual-path with cross-path patterns.

To suppress the interference of irrelevant regions, we further utilize the spatial attention module and the channel attention module. For channel attention, it suppresses the less informative channels by learning channel attention weights in the channels as feature selectors that indicate the importance of each feature channel, channel attention focuses on “what” is meaningful for a given input image. Unlike the channel attention module, the spatial attention module is concerned with “where” the information part is, and as a complement to the channel attention module, spatial attention obtains the importance of each spatial location by learning spatial attention weights. They enable the network to identify key features by their spatial location and thus improve the feature representation of different subtypes.

### Depth feature extraction module

The features learned from the three branches are fused according to their different characteristics. LTTpath1 and LTTpath2 complement each other’s information through additive operations. Since the features extracted from SFEpath are shallow information such as the texture and shape of the breast image, the features extracted from SFEpath are used as complementary information to the fused features of LTTpath1 and LTTpath2. The fusion is superimposed by a concatenation operation to reduce the loss of information.

As shown in **Figure 3A**, the fusion of features from multiple branches by path4 reduces the size of the feature map by half and doubles the number of feature maps, maintaining the complexity of the network layer. The deep features are further extracted by increasing the number of channels and setting stride to 2 to remove the residual connected blocks, and finally, the extracted deep features are used for the classification of breast cancer subtypes.

### Parameter setting

We implemented the proposed framework and conducted experiments using the Pytorch library. The parallel computation uses a GPU-equipped graphics processing unit (NVIDIA GeForce GTX 2060) to accelerate the processing of training and testing. The batch sizes for training and testing were set to 8 and 1, respectively. the maximum epoch time was set to 200, and the initialized learning rate was 0.002, multiplied by 0.95 every 10 epochs. we chose RMSprop as the optimizer for the training phase. Data overfitting is prevented by limiting the square size of kernel weights and using L2 regularization. The whole framework is trained in an end-to-end manner, and the model is trained with a backpropagation algorithm that saves the model parameters that perform best on the validation set. The whole training process takes 1 hour. The cross-entropy function is chosen as the classification loss function.

### Statistical analysis

TP(True Positive):The number of samples judged to be correct among those judged to be positive.

FP(False Positive):The number of misjudgments in samples judged positive.

TN(True Negative)The number of correct samples among those judged negative.

FN(False Negative):The number of judgment errors in samples judged negative.


(1) $$\text{Accuracy}=\frac{TP+TN}{TP+TN+FP+FN}$$

(2) $$\text{Precision}=\frac{TP}{TP+FP}$$

(3) $$\text{Recall=}\frac{TP}{TP+FN}$$

(4) $$F1=\frac{2\times precision\times recall}{precision+recall}$$

For each subtype of disease, we report five metrics, namely: Acc(Accuracy), Pre(Precision), Rec(Recall), F1(F1 score), and AUC(Area under the ROC curve).

## Results

### Comparison of CAMBNET and classical CNN

**Figure 4:**
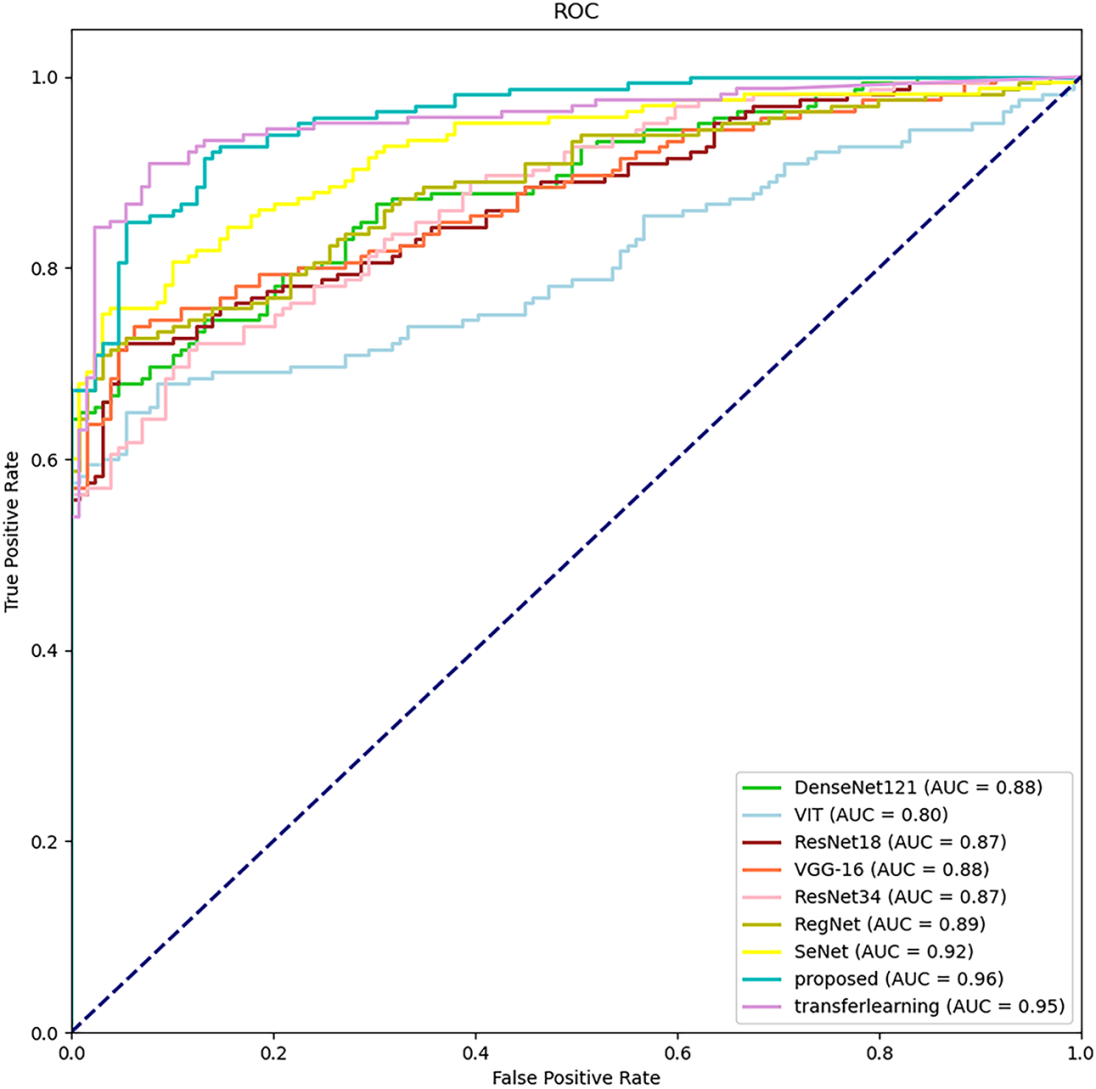
Receiver operating characteristic (ROC) curves of correlation networks in test dataset.

The classification results of different methods according to the evaluation metrics are shown in **Table 3**. we also performed migration learning experiments on our proposed CAMBNET model. We selected 3200 images from the Breakhis database as the training set and 1010 images as the test set to initially train the model. During the training process, the training model parameters with the best classification results were saved, and then the model parameters were reloaded and further trained on the training set of the target dataset we collected. At the same time, this paper performs migration learning while performing real-time data enhancement on each breast MRI image in training. The main implementation method is to perform random rotation and flip along the diagonal of the image. According to **Table 3**, our model achieves the best results in all classification metrics, with an Accuracy of 88.44%, Precision of 88.52%, Recall of 89.11%, and F1 of 88.40%. Meanwhile, after transfer learning and data augmentation, the model can be further improved with 89.46% for Accuracy,89.83% for Precision, 90.35% for Recall, and 89.44% for F1. As shown in **Figure 4**, the CAMBNET model has the best performance with an AUC value of 96%.

**Table 3:** Experimental results of correlation networks on the dataset. Transfer+Aug: transfer learning and Data Augmentation.

Model	Acc (%)	Pre (%)	Rec (%)	F1(%)
Densenet121	78.91	83.77	81.21	78.75
Resnet18	75.51	82.09	78.18	75.14
Resnet34	77.55	77.30	77.63	77.37
Vgg16	76.53	82.14	79.01	76.27
VIT	70.78	71.16	71.47	73.77
SENet	82.65	84.96	84.29	82.64
RegNetY_400MF	78.57	83.20	80.82	78.42
CAMBNET	88.44	88.52	89.11	88.40
Transfer+Aug	**89.46**	**89.83**	**90.35**	**89.44**

Bold marks show that it is the highest value.

### Comparison of CAMBNET with other methods

Previous work has been done to classify breast cancer subtypes using existing machine learning methods or building deep learning models. Tianwen Xie et al (31) used KNN, SVM, and other machine learning methods to classify breast cancer subtypes and achieved good experimental results. Richard Ha et al (27) and Rong Sun et al (32) built models for breast cancer subtypes for classification. We compared our method with the specific methods used in the three papers mentioned above, and the experimental results are shown in **Table 4**. As can be seen from **Table 4**, the accuracy of the traditional machine learning methods is significantly lower than the classification accuracy of the deep learning models, while our model exceeds the previously mentioned model methods in all metrics, which fully illustrates the accuracy of our model.

**Table 4:** Comparison of CAMBNET with other methods.

Model	Acc(%)	Pre (%)	Rec (%)	F1(%)
SVM	66.67	69.63	67.57	66.76
KNN	74.15	74.40	74.15	74.05
Richard Ha et al. (27)	82.31	84.22	83.82	82.31
Rong Sun et al. (32)	84.35	85.85	85.72	84.35
CAMBNET	**88.44**	**88.52**	**89.11**	**88.40**

Bold marks show that it is the highest value.

### Multi-source data testing

We collected and collated 40 images acquired by a 1.5T magnetic resonance scanner (HDXT, GE, USA) and replaced 40 images of the source data set with these 40 1.5T images, thus collating a multi-source data set. The CAMB model has also experimented with multi-source data. The specific experimental results are shown in **Table 5**, from which it can be seen that the indicators of the experiments have decreased. However, the CAMB model still achieves 82.29% accuracy on the multi-source data set, and the experimental results show that the CAMB model has strong robustness. We analyze that this is due to the fact that the model uses cross-attention mechanisms, multi-branch paths, dropout, feature fusion, and other measures to ensure the robustness of the model.

**Table 5:** Multi-source data testing.

Dataset	Acc(%)	Pre (%)	Rec (%)	F1(%)
multi_sources	82.29	82.80	82.29	82.22
single_source	**88.44**	**88.52**	**89.11**	**88.40**

Bold marks show that it is the highest value.

### Effect of age at menarche on molecular subtype classification

**Table 6** shows the effect of the patient’s age at menarche (≤14 and >14 years) on the classification effect of the CAMBNET model (33). The experimental results showed that the younger the age at menarche, the better the model classification effect, and the more significant the classification effect in distinguishing between luminal and non-luminal types. At the age of menarche >14 years, the CAMBNET model classified luminal and non-luminal types with an Accuracy of 82.58%, Precision of 83.06%, Recall of 82.85%, F1 of 82.57%, and AUC of 87.45%. The accuracy of the CAMBNET model in classifying luminal and non-luminal types was 92.37% for Accuracy, 92.42% for Precision, 93.33% for Recall, 92.33% for F1, and 99.95% for AUC for age at menarche ≤14 years. The accuracy was 69.23% in cases with age at menarche >14 years and 88.44% in cases with age at menarche ≤14 years.

**Table 6:** Diagnostic performance of CAMBNet for differentiating molecular subtypes based on menarche age.

Dataset	Acc(%)	Pre (%)	Rec (%)	F1(%)	AUC(%)
Mage_large	82.58	83.06	82.85	82.57	87.45
Mage_small	**92.37**	**92.42**	**93.33**	**92.33**	**99.95**
Mage_large_LuminalA	69.23	63.93	62.46	62.92	56.01
Mage_small_LuminalA	88.44	83.45	65.11	69.35	85.68

Mage_large, luminal and non-luminal at age of menarche >14 years;

Mage_small, luminal and non-luminal at age of menarche ≤ 14 years;

Mage_large_LuminalA,luminalA and others at age of menarche >14 years;

Mage_small_LuminalA,luminalA and others at age of menarche ≤14 years.

Bold marks show that it is the highest value.

### Impact of tumor size on molecular subtype classification

As shown in **Table 7**, we conducted experiments on the effectiveness of the CAMBNET model in different tumor size groups (≤2cm and >2cm). In the classification of luminal and non-luminal types, the CAMBNET model had better performance in differentiating luminal and non-luminal types in the >2 cm group with an AUC of 95.87% and ACC of 88.07%. 89.35% for Precision,85.81% for Recall, and 86.97% for F1. However, in the classification experiment between triple-negative and non-triple-negative types, the CAMBNET model had better discriminatory performance in the <2 cm group with an AUC of 83.45% and ACC of 90.29%. 94.79% for Precision, 70.59% for Recall, and 76.42% for F1.

**Table 7:** Diagnostic performance of CAMBNet for differentiating molecular subtypes based on tumor size.

Dataset	Acc(%)	Pre (%)	Rec (%)	F1(%)	AUC(%)
size_large	88.07	89.35	**85.81**	**86.97**	**95.87**
size_small	82.73	85.24	82.97	82.48	84.62
size_large_TN	86.62	78.88	62.86	66.24	48.45
size_small_TN	**90.29**	**94.79**	70.59	76.42	83.45

size_large, luminal and non-luminal at size of tumor >2cm;

size_small, luminal and non-luminal at size of tumor ≤2cm;

size_large_TN, triple-negative and others at size of tumor >2cm;

size_small_TN, triple-negative and others at size of tumor ≤2cm.

Bold marks show that it is the highest value.

### Visual analysis of CAMBNET

Although the CAMBNET model has achieved high accuracy in breast cancer subtype classification, the lack of visual analysis severely limits its application in realistic tasks. Therefore, we experimentally demonstrate the reliability and feasibility of this method by performing a visual analysis of the CAMBNET model.

**Figure 5:**
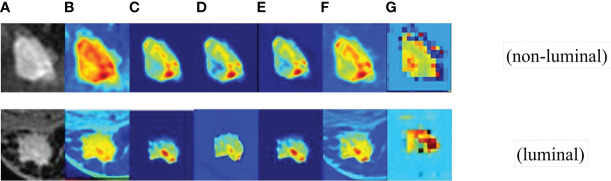
The visual images of feature. **(A)** the feature Source images. **(B)** the first convolutional layer images. **(C)** SFEpath images. **(D)** LTTpath1 images. **(E)** LTTpath2 images. **(F)** the three branches fused images. **(G)** DEFM images.

First, we obtained the visual images of the feature shown in **Figure 5**, and the higher brightness in the feature map indicates higher attention and a higher contribution to the classification performance. Darker pixel regions such as blue indicate a smaller proportion of training and a smaller contribution to the classification performance. As shown in **Figure 5**, the focused regions of FEATURE MAP are consistent with the locations of key lesions that physicians focus on, which demonstrates that the method can well localize image features with clinical diagnostic value and proves the effectiveness of the CAMBNET model in breast cancer subtype classification.

**Figure 6:**
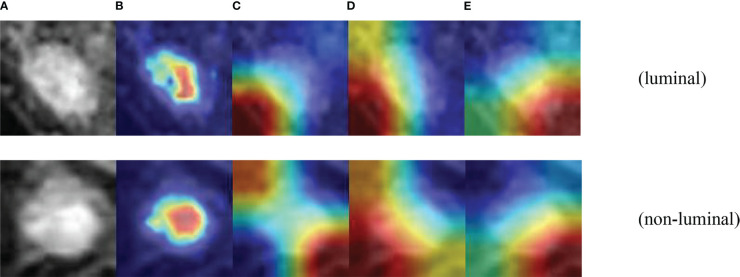
Visualization of CAMB CNN. **(A)** Source images. **(B)** CAMBNet images. **(C)** ResNet34 images. **(D)** DenseNet121 images. **(E)** Vgg16 images.

To further demonstrate the effectiveness of the designed multi-branch attention network, we visualized the learned features with the CAMBNET model and the ResNet34 (34), DenseNet121 (35), Vgg16 (36) networks which have better performance in classification in the classical model by Grad-CAM. Grad-CAM is a gradient-weighted class combining gradient information with the feature mapping activation mapping method. Given an input sample, Grad-CAM first calculates the gradient of the target class for each feature map in the last convolutional layer and performs a global average pooling of the gradients. The importance weight of each feature map is obtained by global average pooling. Then, the weighted activation of the feature maps is calculated based on the importance weights to obtain a gradient-weighted class activation map. The gradient-weighted class activation map can be used to locate the important regions with class discriminative properties in the input samples. The results are shown in **Figure 6**, and we can see that the focus region of our designed multi-branch attention network is mainly on the tumor itself compared with other classical networks. Meanwhile, the focus of other models is often not on the tumor itself but on other non-focus regions. This indicates that compared with other classical models, the CAMBNET model can better learn the features of important regions and focus on the discriminative features between different subtypes, and finally achieve accurate classification of subtypes.

## Discussion

It has been reported that the histological features based on DCE-MRI images of the breast are helpful to differentiate the molecular types of breast cancer. Fan et al. (28) found that the imaging omics model based on DCE-MRI was good at identifying the molecular subtypes of breast cancer. Agner et al. (37) retrospectively analyzed the DCE-MRI images of 76 patients with breast cancer and analyzed the differences between triple-negative breast cancer (TNBC) and other molecular subtypes. Sun et al. (37) retrospectively analyzed the DCE-MRI images of 266 breast cancer patients and used a convolutional neural network (CNN) to distinguish breast cancer subtypes (luminal and non-luminal). The average prediction specificity, accuracy, precision, and area under the ROC curve were 0.958, 0.852, 0.961, and 0.867, respectively. Another study (26) also used a convolutional neural network (CNN) algorithm to predict the molecular subtype of breast cancer based on the MRI features of breast cancer and achieved good diagnostic efficiency. The above study demonstrates the feasibility of using deep learning to classify different molecular subtypes of breast cancer. To further improve the performance of breast cancer subtype classification based on breast MRI images, we propose a new deep network model to extract high level feature information and focus on lesion objects. Experiments conducted on the MRI dataset of 160 clinical breast tumor patients obtained from the Second Hospital of Dalian Medical University showed that the recall, accuracy, precision, and area under the ROC curve of our method were 89.11%, 88.44%, 88.52%, and 96.10% for luminal and non-luminal types. The above experimental results verify the effectiveness of the model, and we used transfer learning and data augmentation for the CAMBNET model to further improve the model’s ability to classify breast cancer subtypes. Among them, Accuracy of 89.46%, Precision of 89.83%, Recall of 90.35%, and F1 of 89.44%. Histopathological analysis of breast cancer has achieved high accuracy in recent years. Chuang Zhu et al. (38) proposed a method for histopathological image classification of breast cancer by combining multiple compact convolutional neural networks (CNN). Mustafa I. Jaber et al. (39) developed a deep learning method for subtype classification of tumors using only breast biopsy tissue sections. Related work achieved high accuracy rates and we compared these models with our model and showed that the results achieved by both are comparable. However, MRI has the advantage of being noninvasive and fast, whereas histopathological images are invasive and also have slower feedback of results, so our work still has a relative advantage. And the interpretability of the machine learning results was achieved through the visual analysis of the CAMBNET model. It shows that the method is reliable and feasible.

TNM staging of breast cancer is of great importance for guiding treatment, evaluating the curative effect, and assessing prognosis (40). Whether the maximum diameter of the tumor is more than 2 cm is the most important index for distinguishing T1 from T2. TNBC has the highest invasiveness and the worst prognosis. Some studies (41) have found that the diameter of the primary tumor of TNBC positively correlates with the axillary lymph node metastasis rate. When the tumor diameter exceeds 2 cm, the ipsilateral axillary lymph node metastasis rate increases by 50% (42, 43). In this study, our model performed best in distinguishing TNBC from NTNBC in the group with tumor diameter ≤ 2 cm. The accuracy is 90.29% and the AUC value is 83.45%, which is helpful for the early diagnosis and treatment of TNBC, improving the prognosis and survival rate of patients. Early age of menarche is one of the risk factors for breast cancer (44). The younger the age of menarche, the earlier a woman is exposed to estrogen. Studies have shown that the earlier the age of menarche, the worse the degree of differentiation and prognosis of breast cancer patients, and the higher the rate of axillary lymph node metastasis (29). Studies have reported that the earlier the age of menarche, the higher the incidence of non-luminal breast cancer and the higher the malignancy, and the worse the prognosis of non-luminal breast cancer compared to luminal breast cancer. For the classification of luminal and non-luminal breast cancer, our results show that the ACC value for menarche age ≤ 14 years is 92.37%, precision is 92.42%, recall is 93.33%, F1 is 92.33%, and AUC is 99.95%. At the same time, we investigated the classification efficiency of our model in luminal type A and non-luminal type A. The results showed that the ACC diagnostic efficiency for menarche age ≤ 14 years was 88.44%, precision 83.45%, recall 65.11%, F1 69.35%, and AUC 85.68%. Our model is more valuable in classifying luminal and non-luminal types of breast cancer patients with menarche age ≤ 14 years.

In this study, there are also some limitations, firstly, only the classification of intraluminal subtypes/non-luminal subtypes was performed in this paper, because the dataset is not sufficient relative to the task of four subtypes. Moreover, annotating such images is laborious and time-consuming, and subsequent work can be performed for weakly supervised or unsupervised learning. Meanwhile, the authors have some textual supplementary information at hand, which can be considered for subsequent experiments to be applied by distillation learning and other methods.

## Conclusion

In summary, the experimental results show that our novel deep learning algorithm based on multi-branch feature fusion and attention mechanism has high accuracy in predicting molecular subtypes of breast cancer, Our model might be more valuable in classifying luminal from non-luminal subtypes for patients with age at menarche ≤14 years. For patients with tumor sizes ≤20 mm, our model could be helpful in more accurately detecting triple-negative breast cancer to improve patient prognosis and survival. So our novel deep learning algorithm has greater potential for future clinical applications. In the near future, we will collect more data to build a larger and more comprehensive breast cancer subtype database to better study the problem of breast cancer subtype classification, aiming to comprehensively assist physicians in the clinical diagnosis and treatment of breast cancer subtypes.

## Data availability statement

The raw data supporting the conclusions of this article will be made available by the authors, without undue reservation.

## Ethics statement

The studies involving human participants were reviewed and approved by Ethics Committee of the Second Hospital of Dalian Medical University Second Hospital of Dalian Medical University. The patients/participants provided their written informed consent to participate in this study.

## Author contributions

LS, HG: Design of this study, writing review and editing. HT: Data processing, design of algorithm, experimental validation, writing of original manuscript. TL, YZ: Design of this study, writing of original manuscript and data collation. JT, YL, CL: Writing of original manuscript, data collation. All authors contributed to this article and approved the manuscript. All authors contributed to the article and approved the submitted version.

## References

[1] Sung H Ferlay J Siegel RL Laversanne M Soerjomataram I Jemal A. Global cancer statistics 2020: GLOBOCAN estimates of incidence and mortality worldwide for 36 cancers in 185 countries. CA Cancer J Clin (2021) 71(3):209–49. doi: 10.3322/caac.21660 33538338

[2] Sun CY Shi JF Fu WQ Zhang X Liu GX Chen WQ. Catastrophic health expenditure and its determinants among households with breast cancer patients in China: A multicentre, cross-sectional survey. Front Public Health (2021) 9:704700. doi: 10.3389/fpubh.2021.704700 34291034 PMC8287064

[3] Brenner DR Weir HK Demers AA Ellison LF Louzado C Shaw A. Projected estimates of cancer in Canada in 2020. CMAJ (2020) 192(9):E199–205. doi: 10.1503/cmaj.191292 PMC7055947 32122974

[4] Szymiczek A Lone A Akbari MR. Molecular intrinsic versus clinical subtyping in breast cancer: A comprehensive review. Clin Genet (2021) 99(5):613–37. doi: 10.1111/cge.13900 33340095

[5] Li X Zhou J Xiao M Zhao L Zhao Y Wang S. Uncovering the subtype-specific molecular characteristics of breast cancer by multiomics analysis of prognosis-associated genes, driver genes, signaling pathways, and immune activity. Front Cell Dev Biol (2021) 9:689028. doi: 10.3389/fcell.2021.689028 34277633 PMC8280810

[6] Zhang X. Molecular classification of breast cancer: Relevance and challenges. Arch Pathol Lab Med (2023) 147(1):46–51. doi: 10.5858/arpa.2022-0070-RA 36136295

[7] Yuan C Jin F Guo X Zhao S Li W Guo H. Correlation analysis of breast cancer DWI combined with DCE-MRI imaging features with molecular subtypes and prognostic factors. J Med Syst (2019) 43(4):83. doi: 10.1007/s10916-019-1197-5 30810823

[8] Yetkin DI Akpinar MG Durhan G Demirkazik FB. Comparison of clinical and magnetic resonance imaging findings of triple-negative breast cancer with non-triple-negative tumours. Pol J Radiol (2021) 86:e269–e76. doi: 10.5114/pjr.2021.106137 PMC8186308 34136044

[9] Zhang Y Zhu Y Zhang K Liu Y Cui J Tao J. Invasive ductal breast cancer: preoperative predict ki-67 index based on radiomics of ADC maps. Radiol Med (2020) 125(2):109–16. doi: 10.1007/s11547-019-01100-1 31696388

[10] Bitencourt A Gibbs P Saccarelli CR Daimiel I Jochelson MS. MRI-Based machine learning radiomics can predict HER2 expression level and pathologic response after neoadjuvant therapy in HER2 overexpressing breast cancer. EBioMedicine (2020) 61:103042. doi: 10.1016/j.ebiom.2020.103042 33039708 PMC7648120

[11] Liu Z Li Z Qu J Zhang R Zhou X Li L. Radiomics of multiparametric MRI for pretreatment prediction of pathologic complete response to neoadjuvant chemotherapy in breast cancer: A multicenter study. Clin Cancer Res (2019) 25(12):3538–47. doi: 10.1158/1078-0432.CCR-18-3190 30842125

[12] Whitney HM Li H Ji Y Liu P Giger ML. Comparison of breast MRI tumor classification using human-engineered radiomics, transfer learning from deep convolutional neural networks, and fusion methods. Proc IEEE Inst Electr Electron Eng. (2020) 108(1):163–77. doi: 10.1109/jproc.2019.2950187 PMC8152568 34045769

[13] Wu Z Qiu J Mu Z Lu W Shi L. Multiparameter MR-based radiomics for the classification of breast cancer molecular subtypes. Int J Radiat OncologyBiologyPhysics (2020) 108(3):e786. doi: 10.1016/j.ijrobp.202007.253

[14] Zhang Z Li Y Wu W Chen H Cheng L Wang S. Tumor detection using deep learning method in automated breast ultrasound. Biomed Signal Process Control (2021) 68:102677. doi: 10.1016/j.bspc.2021.102677

[15] Shkolyar E Jia X Chang TC Trivedi D Mach KE Meng MQ. Augmented bladder tumor detection using deep learning. Eur Urol (2019) 76(6):714–8. doi: 10.1016/j.eururo.2019.08.032 PMC6889816 31537407

[16] Liu WC Li MX Wu SN Tong WL Li AA Sun BL. Using machine learning methods to predict bone metastases in breast infiltrating ductal carcinoma patients. Front Public Health (2022) 10:922510. doi: 10.3389/fpubh.2022.922510 35875050 PMC9298922

[17] Guo YY Huang YH Wang Y Huang J Lai QQ Li YZ. Breast MRI tumor automatic segmentation and triple-negative breast cancer discrimination algorithm based on deep learning. Comput Math Methods Med (2022) 2022:2541358. doi: 10.1155/2022/2541358 36092784 PMC9453096

[18] Xing F Xie Y Yang L. An automatic learning-based framework for robust nucleus segmentation. IEEE Trans Med Imaging (2016) 35(2):550–66. doi: 10.1109/TMI.2015.2481436 26415167

[19] Henschel L Kugler D Reuter M. FastSurferVINN: Building resolution-independence into deep learning segmentation methods-a solution for HighRes brain MRI. Neuroimage (2022) 251:118933. doi: 10.1016/jneuroimage.2022.118933 35122967 PMC9801435

[20] Al-Garaawi N Ebsim R Alharan AFH Yap MH. Diabetic foot ulcer classification using mapped binary patterns and convolutional neural networks. Comput Biol Med (2021) 140:105055. doi: 10.1016/j.compbiomed.2021.105055 34839183

[21] Ding S Wu Z Zheng Y Liu Z Yang X Yang X. Deep attention branch networks for skin lesion classification. Comput Methods Programs Biomed (2021) 212:106447. doi: 10.1016/j.cmpb.2021.106447 34678529

[22] Leong YS Hasikin K Lai KW Mohd Zain N Azizan MM. Microcalcification discrimination in mammography using deep convolutional neural network: Towards rapid and early breast cancer diagnosis. Front Public Health (2022) 10:875305. doi: 10.3389/fpubh.2022.875305 35570962 PMC9096221

[23] Pawar SD Sharma KK Sapate SG Yadav GY Alroobaea R Alzahrani SM. Multichannel DenseNet architecture for classification of mammographic breast density for breast cancer detection. Front Public Health (2022) 10:885212. doi: 10.3389/fpubh.2022.885212 35548086 PMC9081505

[24] Jiang M Zhang D Tang SC Luo XM Chuan ZR Lv WZ. Deep learning with convolutional neural network in the assessment of breast cancer molecular subtypes based on US images: a multicenter retrospective study. Eur Radiol (2021) 31(6):3673–82. doi: 10.1007/s00330-020-07544-8 33226454

[25] Li C Huang H Chen Y Shao S Chen J Wu R. Preoperative non-invasive prediction of breast cancer molecular subtypes with a deep convolutional neural network on ultrasound images. Front Oncol (2022) 12:848790. doi: 10.3389/fonc.2022.848790 35924158 PMC9339685

[26] Zhang Y Chen JH Lin Y Chan S Zhou J Chow D. Prediction of breast cancer molecular subtypes on DCE-MRI using convolutional neural network with transfer learning between two centers. Eur Radiol (2021) 31(4):2559–67. doi: 10.1007/s00330-020-07274-x PMC8547260 33001309

[27] Ha R Mutasa S Karcich J Gupta N Pascual Van Sant E Nemer J. Predicting breast cancer molecular subtype with MRI dataset utilizing convolutional neural network algorithm. J Digit Imaging. (2019) 32(2):276–82. doi: 10.1007/s10278-019-00179-2 PMC6456631 30706213

[28] Fan M Li H Wang S Zheng B Zhang J Li L. Radiomic analysis reveals DCE-MRI features for prediction of molecular subtypes of breast cancer. PloS One (2017) 12(2):e0171683. doi: 10.1371/journal.pone.0171683 28166261 PMC5293281

[29] Orgeas CC Hall P Rosenberg LU Czene K. The influence of menstrual risk factors on tumor characteristics and survival in postmenopausal breast cancer. Breast Cancer Res (2008) 10(6):R107. doi: 10.1186/bcr2212 19087323 PMC2656904

[30] Hu J Shen L Sun G. (2018). Squeeze-and-Excitation networks, in: 2018 IEEE/CVF Conference on Computer Vision and Pattern Recognition, Salt Lake City, UT, USA. pp. 7132–41. doi: 10.1109/CVPR.2018.00745

[31] Xie T Wang Z Zhao Q Bai Q Zhou X. Machine learning-based analysis of MR multiparametric radiomics for the subtype classification of breast cancer. Front Oncol (2019) 9:505. doi: 10.3389/fonc.2019.00505 31259153 PMC6587031

[32] Sun R Meng Z Hou X Chen Y Yang Y Huang G. Prediction of breast cancer molecular subtypes using DCE-MRI based on CNNs combined with ensemble learning. Phys Med Biol (2021) 66(17):175009. doi: 10.1088/1361-6560/ac195a 34330117

[33] Bravi F Decarli A Russo AG. Risk factors for breast cancer in a cohort of mammographic screening program: a nested case-control study within the FRiCaM study. Cancer Med (2018) 7(5):2145–52. doi: 10.1002/cam4.1427 PMC5943434 29654663

[34] He K Zhang X Ren S Sun J. Deep residual learning for image recognition in proc. CVPR (2016), 770–8. doi: 10.1109/CVPR.2016.90

[35] Huang G Liu Z Laurens VDM Weinberger KQ. (2017). Densely connected convolutional networks, in: 2017 IEEE Conference on Computer Vision and Pattern Recognition (CVPR), . pp. 2261–269. doi: 10.1109/CVPR.2017.243

[36] Simonyan K Zisserman A. Very deep convolutional networks for Large-scale image recognition. Computer Science (2014). doi: 10.48550/arXiv.1409.1556

[37] Agner SC Rosen MA Englander S Tomaszewski JE Feldman MD Zhang P. Computerized image analysis for identifying triple-negative breast cancers and differentiating them from other molecular subtypes of breast cancer on dynamic contrast-enhanced MR images: A feasibility study. Radiology (2014) 272(1):91–9. doi: 10.1148/radiol.14121031 PMC4263619 24620909

[38] Zhu C Song F Wang Y Dong H Guo Y Liu J. Breast cancer histopathology image classification through assembling multiple compact CNNs. BMC Med Inform Decis Mak. (2019) 19(1):198. doi: 10.1186/s12911-019-0913-x 31640686 PMC6805574

[39] Jaber MI Song B Taylor C Vaske CJ Benz SC Rabizadeh S. A deep learning image-based intrinsic molecular subtype classifier of breast tumors reveals tumor heterogeneity that may affect survival. Breast Cancer Res (2020) 22. doi: 10.1186/s13058-020-1248-3 PMC6988279 31992350

[40] Singletary SE Connolly JL. Breast cancer staging: working with the sixth edition of the AJCC cancer staging manual. CA Cancer J Clin (2010) 56(1):37–47. doi: 10.3322/canjclin.56.1.37 16449185

[41] Tamimi RM Colditz GA Hazra A Baer HJ Hankinson SE Rosner B. Traditional breast cancer risk factors in relation to molecular subtypes of breast cancer. Breast Cancer Res Treat (2012) 131(1):159–67. doi: 10.1007/s10549-011-1702-0 PMC3237947 21830014

[42] Kahn HJ Hanna WM Chapman JA Trudeau ME Lickley HL Mobbs BG. Biological significance of occult micrometastases in histologically negative axillary lymph nodes in breast cancer patients using the recent American joint committee on cancer breast cancer staging system. Breast J (2006) 12(4):294–301. doi: 10.1111/j.1075-122X.2006.00267.x 16848838

[43] Conlin AK Seidman AD. Beyond cytotoxic chemotherapy for the first-line treatment of HER2-negative, hormone-insensitive metastatic breast cancer: current status and future opportunities. Clin Breast Cancer (2008) 8(3):215–23. doi: 10.3816/CBC.2008.n.024 18650151

[44] Yang XR Sherman ME Rimm DL Lissowska J Brinton LA Peplonska B. Differences in risk factors for breast cancer molecular subtypes in a population-based study. Cancer Epidemiol Biomarkers Prev (2007) 16(3):439–43. doi: 10.1158/1055-9965.EPI-06-0806 17372238

